# Impact of Short-Term Dietary Restriction Combined with Amaranth and Canola Oil Supplementation on Salivary Adipokines in Adults with Obesity

**DOI:** 10.3390/nu18040628

**Published:** 2026-02-14

**Authors:** Marzena Helwich, Dominika Kanikowska, Wojciech Eliasz, Alina Kanikowska, Rafał Rutkowski, Małgorzata Moszak, Aldona Juchacz, Ewelina Swora-Cwynar, Marian Grzymisławski, Elżbieta Paszyńska, Anna Surdacka

**Affiliations:** 1 Department of Conservative Dentistry, Poznan University of Medical Sciences, 60-712 Poznan, Poland; marzena.dabrowska@ump.edu.pl (M.H.); annasurd@ump.edu.pl (A.S.); 2 Department of Integrated Dentistry and Endodontics, Poznan University of Medical Sciences, 60-712 Poznan, Poland; paszynska@ump.edu.pl; 3 Department of Pathophysiology, Poznan University of Medical Sciences, 60-712 Poznan, Poland; dkanikowska@ump.edu.pl (D.K.); rrutkowski@ump.edu.pl (R.R.); 4 Department of Gastroenterology, Dietetics and Internal Disease, Poznan University of Medical Science, 60-712 Poznan, Poland; akanikowska@ump.edu.pl (A.K.); ajuchacz@wcpit.org (A.J.); eswora@ump.edu.pl (E.S.-C.); mariangrzym@ump.edu.pl (M.G.); 5 Department of Obesity and Metabolic Disorder Treatment and Clinical Dietetics, Poznan University of Medical Sciences, 60-712 Poznan, Poland; mmoszak@ump.edu.pl

**Keywords:** obesity, saliva, amaranth oil, canola (rapeseed) oil, calorie restriction diet, PAI-1, TNF R1, serpin A12

## Abstract

**Background:** Adipose tissue is a crucial endocrine organ, and obesity, due to its associated chronic inflammation and oxidative stress, disrupts adipokine secretion. These adipokines can be detected not only in blood but also in saliva. Dietary changes are a crucial part of managing obesity, encompassing a balanced diet, increased physical activity, and lifestyle modifications. Moreover, adding functional foods like amaranth and canola oils, recognized for their health benefits, may further improve metabolic and inflammatory health. These products have anti-inflammatory effects and may help reduce the pro-inflammatory activity of adipose tissue, thereby improving systemic and oral health. The study aimed to assess the impact of a 3-week calorie-restricted diet, supplemented with canola or amaranth oil on salivary adipokines, i.e., serpin A12, plasminogen activator inhibitor-1 (PAI-1), and tumor necrosis factor receptor-1 (TNF-R1), pH, and salivary flow in obese patients. **Methods:** A total of 115 adults with obesity (BMI > 30 kg/m^2^) were enrolled and placed on a 3-week calorie-restricted diet. The study group (n = 44) received additional supplementation: 21 participants received 20 mL of canola oil daily, and 23 received 20 mL of amaranth oil. The control group (n = 71) followed the same calorie-restricted diet without oil supplementation. Non-stimulated saliva was collected twice, for 20 min each time, before and after the intervention, to evaluate flow rate, pH, and concentrations of serpin A12, PAI-1, and TNF-R1. Concentrations were measured using enzyme-linked immunosorbent assay (ELISA). **Results:** An increase in saliva flow rate was observed in patients supplemented with amaranth oil (*p* = 0.0367). Both the amaranth oil and canola oil groups showed a significant rise in salivary pH (*p* = 0.0425). Across all participants, the 3-week calorie-restricted diet resulted in a reduction in salivary PAI-1 (*p* = 0.0339), serpin A12 (*p* = 0.0001), and TNF-R1 (*p* = 0.0058). **Conclusions:** The 3-week calorie-restricted diet contributed to a decrease in the concentration of adipokines in saliva. The low-calorie diet, combined with supplementation of amaranth and canola oils, increased salivary flow and resulted in higher pH values, indicating greater alkalinity.

## 1. Introduction

Obesity remains one of the most serious global health challenges of the 21st century. According to the World Health Organization, more than 2.5 billion adults were overweight in 2022, including approximately 890 million with obesity, and the prevalence continues to rise across all age groups [1]. Modeling studies predict that, if current trends persist, over half of the world’s population will be overweight or obese by 2035. Among adults, the global prevalence of obesity has more than doubled over the same period, reaching approximately 16% in 2022. Excess body weight appears to be associated with higher mortality than underweight [2].

Obesity is a major risk factor for cardiovascular disease, type 2 diabetes, hypertension, and several types of cancer [3]. Governmental and public-health initiatives that promote healthy dietary habits and regular physical activity can reduce morbidity, mortality, and healthcare costs. However, despite these efforts, the global burden of obesity continues to grow, underscoring the need for innovative diagnostic and therapeutic strategies [4].

Once regarded merely as an energy reservoir, adipose tissue is now recognized as a metabolically active endocrine organ that secretes numerous cytokines and hormones, collectively known as adipokines, which regulate appetite, insulin sensitivity, lipid and glucose metabolism, and inflammatory responses [5,6]. Obesity induces a chronic, low-grade inflammatory state and oxidative stress [7], characterized by elevated secretion of pro-inflammatory adipokines such as tumor necrosis factor-α (TNF-α), plasminogen activator inhibitor-1 (PAI-1), and serpin A12 [8,9]. This dysregulated adipokine profile contributes to insulin resistance and the development of metabolic syndrome and cardiovascular disease [5,6].

Adipokines and other bioactive molecules are detectable not only in blood but also in saliva. Saliva represents a non-invasive and cost-effective alternative for assessing systemic inflammation and metabolic status [10]. Recent reviews have highlighted its potential for detecting metabolic disorders, including obesity and insulin resistance [11]. Proteomic analyses indicate that weight loss and lifestyle interventions lead to measurable alterations in salivary proteins and inflammatory mediators [12], supporting the use of salivary biomarkers for early disease detection and longitudinal monitoring. Dietary modification remains a cornerstone of obesity management. A growing body of evidence supports replacing animal fats with plant-derived oils rich in unsaturated fatty acids and phytosterols to reduce systemic inflammation and improve lipid profiles [13,14]. Among these, canola (also known as rapeseed oil) and amaranth oil have garnered particular attention for their cardioprotective and anti-inflammatory properties. Canola (*Brassica napus* L.)

oil is rich in monounsaturated and polyunsaturated fatty acids, including omega-3s, and has been associated with reductions in low-density lipoprotein cholesterol (LDL-C), apolipoprotein B, and circulating insulin [15]. Experimental studies indicate that canola oil modulates hepatic lipid metabolism via 5’AMP-activated protein kinase (AMPK) activation and beneficially alters gut microbiota composition [16]. Amaranth (*Amaranthus cruentus* L.) oil is likewise notable for its high squalene content and favorable fatty acid profile, which may help reduce oxidative stress and inflammation [17]. Both oils have been proposed as functional food ingredients capable of attenuating the pro-inflammatory activity of adipose tissue, thereby improving systemic metabolic health, and potentially oral health as well [18].

Our study evaluates whether the addition of amaranth oil or canola oil to a standardized, calorie-restricted diet influences salivary biomarkers and oral parameters in patients with obesity, treated under controlled hospital conditions. We hypothesize that, owing to their reported anti-inflammatory properties, amaranth oil and canola oil may beneficially affect salivary flow, pH, and concentrations of serpin A12, PAI-1, and TNF-R1 during the three-week dietary intervention.

## 2. Materials and Methods

A total of 115 patients with obesity, defined as a body mass index (BMI) greater than 30 kg/m^2^, (n = 115, including 67 females and 48 males), aged 20–65 years, were included in the study. Participants were treated at the Department of Gastroenterology, Dietetics, and Internal Diseases, Poznań University of Medical Sciences, and admitted to the Greater Poland Center of Pulmonology and Thoracic Surgery (Ludwikowo ward). All participants were hospitalized throughout the entire intervention period and remained under standardized conditions, including the same medical supervision, uniform hospital meals, and structured dietary counseling provided by the same clinical dietitians. Hospitalization was part of a structured inpatient obesity treatment program fully covered by the national health insurance system, with no personal costs to patients. This inpatient setting enabled complete standardization of lifestyle conditions, including supervised dietary intake, structured physical activity, and consistent medical oversight, thereby minimizing inter-individual variability and maximizing protocol adherence. Physical activity was supervised and identical for all participants throughout the intervention. The standardized exercise protocol included: (1) daily breathing and mobility exercises, (2) aerobic training consisting of Nordic walking or cycling performed at 50–70% of maximum heart rate (HRmax), and (3) resistance training. This uniform physical activity regimen was integrated into the calculation of total daily energy expenditure using the Harris-Benedict formula, ensuring that caloric prescriptions appropriately accounted for exercise-related energy expenditure and maintained comparable energy balance across all groups.

Each patient followed a three-week calorie-restricted dietary program. Exclusion criteria included overt diabetes, a history of bariatric surgery, and pregnancy. The sample size was not determined a priori. The number of participants resulted from the availability of eligible hospitalized patients undergoing obesity treatment during the recruitment period. A post hoc power analysis was performed based on the strongest within-group response observed among the salivary adipocytokines. For serpin A12 in the amaranth-oil subgroup (n = 23), the observed change corresponded to a medium effect size (Cohen’s dz ≈ 0.49), yielding an achieved statistical power of approximately 80% at α = 0.05. This indicates that the study was adequately powered to detect moderate within-group effects.

Each eligible and consenting participant was assigned a unique code as an identifier. The study population was randomly assigned to 3 treatment groups. Participants and investigators were blinded to randomization and group allocation. Patients who consented to receive additional oil supplementation were assigned to this group. The study group comprised 44 patients (n = 44). To minimize potential selection bias, all groups were treated in the same ward and followed identical therapeutic protocols. Dietary intake was fully controlled through hospital-provided meals, ensuring comparable caloric content, macronutrient composition, and meal timing across all groups. All participants followed an individualized low-calorie diet providing 1600–2500 calories per day, with specific caloric prescriptions determined based on each individual’s basal metabolic rate and activity level. The intervention groups received their assigned functional oils (canola or amaranth) as part of this dietary regimen, while the control group maintained the low-calorie diet without functional oil supplementation. The hypocaloric diet was based on a 25–30% reduction in caloric intake compared to total energy requirement. The total daily energy requirement was calculated using the Harris-Benedict formula applied to actual body weight and adjusted for physical activity level index. To accurately determine baseline total energy expenditure and dietary energy intake, subjects completed a 3-day dietary intake assessment before the intervention (three 24 h dietary recalls). During the intervention, all subjects received standardized meals prepared by a single catering service, ensuring consistent macronutrient composition across participants: 20% of total energy from protein, 25–30% from fat, and 50–55% from carbohydrates. Daily fiber intake exceeded 25 g.

The study group (n = 44) was subdivided according to the type of supplementation:

- Canola oil (subgroup A), (n = 21): participants received 20 mL of canola oil daily.
- Amaranth oil (subgroup B), (n = 23): participants received 20 mL of amaranth oil daily.

In both subgroups, the additional oil replaced 20 g of dietary fat to maintain isocaloric intake. The 20 mL dose was chosen based on common daily dietary fat recommendations and previous nutritional intervention studies involving similar amounts of plant oils [19]. Adherence to supplementation was monitored daily by nursing staff during inpatient meal distribution.

The control group consisted of 71 patients (n = 71) who followed the same calorie-restricted diet without receiving oil supplementation, while remaining under identical inpatient conditions. Baseline clinical characteristics were assessed to confirm comparability between groups at the start of the study (Table 1).

Although the Kruskal–Wallis test indicated a nominally significant difference in waist circumference (*p* = 0.026), none of the pairwise comparisons remained significant after Bonferroni correction for multiple testing: Canola vs. Amaranth: *p* = 0.12 (not significant), Canola vs. Control: *p* = 0.95 (not significant), Amaranth vs. Control: *p* = 0.52 (not significant)

This suggests appropriate baseline matching across all groups, with the two intervention groups (canola and amaranth) being particularly well-matched to each other across all anthropometric measurements.

Non-stimulated saliva was collected twice, for 20 min each, before and after the intervention, to evaluate flow rate, pH, and concentrations of serpin A12, PAI-1, and TNF-R1. The adipokine panel (PAI-1, serpin A12, TNF-R1) was selected a priori based on pathophysiological relevance to obesity and analytical feasibility in saliva. These biomarkers represent key pathways linking obesity to metabolic dysfunction: PAI-1 reflects endothelial dysfunction and thrombotic risk, serpin A12 is involved in metabolic regulation and insulin sensitivity, and TNF-R1 mediates chronic inflammation. All three adipokines have been previously validated as detectable in saliva and provide complementary insights into obesity-related inflammatory, metabolic, and vascular pathophysiology. Concentrations were determined using enzyme-linked immunosorbent assay (ELISA). Statistical analyses were conducted using Statistica 13 (StatSoft, Tulsa, OK, USA).

The study described in our manuscript was not prospectively registered in a clinical trial registry. At the time of project initiation, it was designed and conducted as a short-term, single-center nutritional intervention without drug administration or patient-relevant clinical endpoints. All procedures were approved by the Ethics Committee of Poznań University of Medical Sciences (approval No. 247/19). Under the local regulations and institutional practice at that time, such physiological studies were not routinely registered as clinical trials. All participants provided written informed consent, and the study was conducted in full accordance with the Declaration of Helsinki and institutional guidelines.

Salivary concentrations of serpin A12, plasminogen activator inhibitor-1 (PAI-1), and tumor necrosis factor receptor-1 (TNF-R1) were determined using enzyme-linked immunosorbent assay (ELISA) with DuoSet^®^ Immunoassay Development Kits (R&D Systems, Minneapolis, MN, USA).

The assay sensitivities were as follows: PAI-1, 140 pg/mL; serpin A12, 10.36 pg/mL; and TNF-R1, 6.95 pg/mL. The reported sensitivities represent limits of detection (LOD), the lowest concentrations distinguishable from zero. Quantification near the LOD is valid when values fall within the calibrated standard curve, as per standard ELISA methodology. Salivary biomarker concentrations are physiologically lower than serum levels; thus, PAI-1 values approaching the LOD are expected. All samples were analyzed per manufacturer protocol, with concentrations calculated from the standard curve. No values were excluded based on proximity to the LOD.

Unstimulated whole saliva was used for analysis. To minimize diurnal variation in composition and volume, all collections were performed in the morning while participants were fasting or had abstained from food for at least 2 h. Saliva was collected twice: the first sample on the day of hospital admission, before the dietary intervention, and the second after 3 weeks of intervention.

During and after collection, tubes were kept on ice. Participants were seated comfortably in a relaxed, slightly forward-leaning position, allowing saliva to drain passively into a graduated collection tube placed in an ice-filled container. Each collection lasted 20 min, and salivary flow rate was calculated in mL/min.

Immediately after collection, samples were kept on ice until centrifugation. Saliva was transported under isothermal conditions to the Department of Pathophysiology, Poznań University of Medical Sciences, where it was centrifuged at 2000 rpm for 10 min and stored at −80 °C until analysis. This collection protocol is considered reliable and appropriate for the quantification of salivary adipokines.

**Figure 1:**
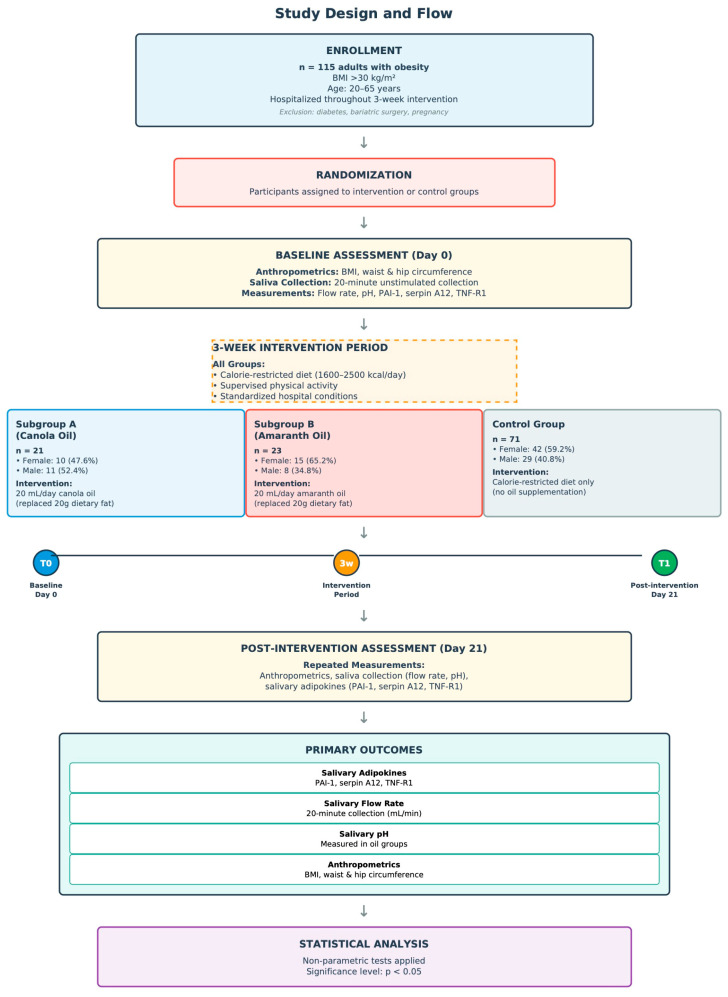
The complete study design, including participant flow and outcome measurements.

Statistical analyses were performed using appropriate parametric and non-parametric tests depending on data distribution and measurement type. Normality of data distribution was assessed using the Shapiro–Wilk test. As data did not follow a normal distribution, non-parametric tests were applied. For salivary flow rate, between-subgroup comparisons were performed using the Mann–Whitney U test. Comparative analysis of pH values between subgroups were conducted using the Mann–Whitney U test. Comparative analysis regarding adipocytokine concentration between subgroups was performed using the Mann–Whitney U test. The complete study design, including participant flow and outcome measurements, is summarized in Figure 1.

## 3. Results

Regarding anthropometric measurements, all three groups demonstrated reductions in anthropometric parameters following the intervention. The amaranth group showed mean reductions of 1.91 kg/m^2^ in BMI (4.62%), 3.88 cm in waist circumference (3.17%), and 4.26 cm in hip circumference (3.25%). The canola group exhibited mean reductions of 1.27 kg/m^2^ in BMI (3.32%), 3.21 cm in waist circumference (2.46%), and 1.87 cm in hip circumference (1.45%). The control group showed mean reductions of 2.80 kg/m^2^ in BMI (5.68%), 5.80 cm in waist circumference (4.16%), and 7.10 cm in hip circumference (5.00%).

Results of biochemical saliva analyses are summarized in Table 2.

In our cohort, salivary PAI-1 concentrations decreased by 21.22% after the three-week calorie-restricted diet in all participants with obesity (n = 115): 97.35 ± 77.03 pg/mL post-intervention versus 123.58 ± 105.30 pg/mL at baseline (*p* = 0.0339). There were no differences in salivary PAI-1 levels between subgroups A (canola oil) and B (amaranth oil) (Table 2), nor when compared to the non-supplemented control group. When analyzed by individual subgroups, PAI-1 concentrations showed numerical reductions in all groups, but these changes did not reach statistical significance (Subgroup A: *p* = 0.5922; Subgroup B: *p* = 0.2361; Control: *p* = 0.0877). However, when all participants were pooled to assess the overall effect of calorie restriction, a statistically significant reduction in salivary PAI-1 was observed (*p* = 0.0339), indicating that the intervention produced a modest but consistent downward trend across the cohort.

In the entire cohort, serpin A12 levels decreased by 39.86% from 454.69 ± 583.94 pg/mL at baseline to 273.43 ± 405.89 pg/mL post-intervention (*p* = 0.0001). Among supplemented participants (canola + amaranth; n = 44), concentrations declined by 30.40% from 385.19 ± 535.69 pg/mL to 268.09 ± 457.62 pg/mL (*p* = 0.0055), whereas in controls by (n = 71) they fell by 42.32% from 479.76 ± 611.66 pg/mL to 276.74 ± 373.67 pg/mL (*p* = 0.0014). In subgroup analyses, no change was observed for canola oil, while amaranth oil was associated with a reduction by 42.08%; *p* = 0.0037 (Table 2). In summary, weight loss was linked to a decrease in salivary serpin A12 levels, with a trend toward a further reduction in the amaranth oil group. However, there were no significant differences between the amaranth and canola oil groups.

In our cohort of adults with obesity (n = 115), salivary TNF-R1 concentrations decreased by 16.46% following the three-week calorie-restricted diet (343.65 ± 267.10 pg/mL post-intervention vs. 411.38 ± 292.74 pg/mL at baseline; *p* = 0.0058). In the supplemented group (canola + amaranth; n = 44), no significant change in salivary TNF-R1 was observed (465.44 ± 311.33 pg/mL post-intervention vs. 514.27 ± 307.03 pg/mL at baseline. By contrast, in the control group (no supplementation; n = 71), TNF-R1 levels decreased significantly by 22.86% (268.17 ± 203.83 pg/mL post-intervention vs. 347.62 ± 266.23 pg/mL at baseline; *p* = 0.0188).

Subgroup analyses showed that canola oil (A; n = 21) was not associated with a change, whereas amaranth oil (B; n = 23) caused a reduction in TNF-R1 concentrations (*p* = 0.0074; Table 2). Post-intervention TNF-R1 levels also differed between the canola and amaranth subgroups (*p* = 0.0024). These findings align with previous reports of elevated TNF-R1 concentrations in obesity and their decrease following dietary restriction.

Salivary flow was measured before and after the intervention. In the study group, the flow rate increased significantly after the calorie-restricted diet (*p* = 0.0230), while no significant change was observed in the control group (*p* = 0.6831).

Moreover, an increase in salivary flow rate was observed in the subgroup supplemented with amaranth oil (subgroup B; *p* = 0.0367), while no difference was noted in the canola-oil subgroup (subgroup A; *p* = 0.3139). There were no differences in salivary flow rate between subgroups A and B, either before (*p* = 0.7714) or after (*p* = 0.9907) the dietary restriction. The average salivary pH in the study group increased from 6.83 ± 0.47 before the dietary restriction to 7.06 ± 0.46 after the intervention (*p* = 0.0425), showing a higher salivary pH after the calorie-restricted diet (Table 3). In the amaranth-oil subgroup (B), salivary pH rose from 6.74 ± 0.47 to 6.99 ± 0.49, while in the canola-oil subgroup (A), it went from 6.92 ± 0.47 to 7.13 ± 0.43. These within-group changes did not reach statistical significance (Table 3). No differences were observed between subgroups A and B either before (*p* = 0.1835) or after (*p* = 0.4463) the dietary restriction. (Table 3).

## 4. Discussion

This study examined the effects of a three-week calorie-restricted program, supplemented with either canola or amaranth oil, on salivary adipokines in adults with obesity. To our knowledge, it is the first investigation to evaluate salivary concentrations of PAI-1, serpin A12, and TNF-R1 following dietary interventions with functional oils. The study design integrated standardized nutrition, controlled physical activity, and non-invasive biomarker assessment. Certain methodological aspects of the randomization process merit acknowledgment. While the initial study design included random assignment to treatment groups with blinding of participants and investigators, the allocation process was partially compromised by allowing patients who specifically consented to receive additional oil supplementation to be assigned to the intervention groups. This voluntary selection introduces potential allocation bias, as participants willing to undertake dietary supplementation may differ systematically from those in the control group in terms of motivation, health consciousness, or adherence patterns. Such self-selection can confound the interpretation of treatment effects and limit the generalizability of findings. Although efforts were made to minimize selection bias by treating all groups in the same ward under identical therapeutic protocols, these measures cannot fully eliminate the inherent differences introduced by non-random allocation. True randomization with concealed allocation would have strengthened the study’s internal validity and allowed for more robust causal inferences regarding the intervention effects. The intervention significantly reduced salivary concentrations of PAI-1, serpin A12, and TNF-R1, indicating a decrease in both systemic and local inflammatory activity. The decline in all three biomarkers confirms that short-term caloric restriction can beneficially modulate the salivary inflammatory profile. Supplementation with amaranth oil appeared to enhance these effects, particularly for serpinA12 and TNF-R1, whereas canola oil showed no additional influence.

PAI-1 is a serine-protease inhibitor (serpin) and a key adipokine that regulates fibrinolysis by inhibiting tissue-type and urokinase-type plasminogen activators. In obesity, especially visceral obesity, PAI-1 expression in adipose tissue and circulating levels are typically elevated, reflecting adipose dysfunction and chronic low-grade inflammation. Increased PAI-1 is linked to insulin resistance, metabolic syndrome, and a prothrombotic, higher cardiovascular-risk profile. At baseline, participants exhibited elevated salivary PAI-1, consistent with findings by Lehmann-Kalata et al., who demonstrated that individuals with obesity have higher salivary concentrations of PAI-1, serpin A12, and TNF-R1 than those with normal weight. After three weeks of dietary restriction, PAI-1 levels decreased (*p* = 0.0339) [20]. This observation aligns with experimental work by Wang et al., who reported that PAI-1 aggravates adipose-tissue dysfunction through macrophage infiltration, while pharmacological inhibition of PAI-1 improves metabolic parameters and reduces body weight [21]. Similarly, Levine et al. [22] showed that PAI-1 suppression stimulates lipolysis and weight loss in obese mice. The present findings therefore support the role of PAI-1 as a modifiable biomarker that responds to energy restriction and reflects improved adipose-tissue function.

Another parameter assessed in this study was serpin A12 (vaspin), a member of the serine protease inhibitor family. This adipokine exhibits upregulated expression in adipose tissue in obesity, and its circulating levels increase proportionally with BMI [14,15,16].

Serpin A12 levels decreased across all groups, with the greatest reduction seen in the amaranth-oil subgroup. This aligns with earlier studies showing that serpin A12 is upregulated in obesity and insulin resistance as a compensatory mechanism, and that its levels tend to decline as metabolic health improves with weight loss. Kurowska et al. and Wang et al. reported reductions in serpin A12 following dietary restriction, and similar patterns have been observed in serum and adipose tissue [17,19]. Experimental research also shows that overexpression of serpin A12 can protect against excessive weight gain and boost energy expenditure [9], while deficiency is linked to faster metabolic decline [7,20]. The salivary findings in this study reflect these systemic observations, suggesting saliva may be a useful, non-invasive marker of adipose tissue signals and metabolic stress resolution.

The final parameter assessed was tumor necrosis factor-α (TNF-α), a cytokine involved in immune regulation and inflammatory signaling. TNF-α exerts its effects through two receptors, TNF-R1 and TNF-R2, with TNF-R1 being expressed on nearly all nucleated cells. In obesity, circulating TNF-α and its soluble receptors are frequently elevated, contributing to a chronic pro-inflammatory milieu that may also increase susceptibility to oral inflammation.

TNF-R1 concentrations decreased by about 16.46% in the total cohort (*p* = 0.0058), with the most pronounced effect in the amaranth-oil group (*p* = 0.0074). This trend corresponds with reports identifying TNF-R1 as a key salivary biomarker elevated in metabolically unhealthy obesity [8] and with the diagnostic value of combined TNF-R1 × serpin A12 measurements proposed by Zyśk et al. [23]. A similar decline in soluble TNF-R1 was also observed following dietary restriction in endurance athletes [24]. Collectively, these results support the hypothesis that salivary TNF-R1 mirrors systemic inflammatory activity and responds dynamically to nutritional interventions.

A notable feature of our data is the substantial inter-individual heterogeneity in both baseline adipokine concentrations and treatment responses, as reflected in the high standard deviations relative to mean values. This variability is characteristic of obesity research and reflects the biological complexity of obese populations, which differ in metabolic phenotype, degree of insulin resistance, visceral versus subcutaneous fat distribution, inflammatory status, and individual responsiveness to dietary interventions [5]. The observed changes therefore represent average group trends, with considerable individual variation in response magnitude and direction. For PAI-1, this heterogeneity combined with modest effect sizes and limited subgroup sample sizes resulted in non-significant within-group changes, despite a statistically significant reduction in the pooled analysis. This suggests that calorie restriction may produce small to moderate effects on PAI-1 that require larger samples to detect reliably within subgroups. The more robust effects observed for serpin A12 and TNF-R1, which achieved statistical significance in multiple subgroups, likely reflect larger effect sizes or lower baseline variability for these biomarkers.

The heterogeneity observed in our study underscores the need for future research to identify predictors of treatment response and characterize metabolic subphenotypes within obese populations. Factors such as baseline inflammatory status, insulin resistance severity, adipose tissue distribution, genetic polymorphisms in adipokine genes, and gut microbiome composition may influence individual responses to dietary interventions. Personalized nutrition approaches that account for individual metabolic profiles may ultimately prove more effective than one-size-fits-all dietary recommendations. An additional limitation concerns salivary pH measurements, which were collected only in the oil-supplemented groups (canola and amaranth) and not in the control group. This was a design decision made to focus resources on the primary research question regarding functional oil effects on salivary adipocytokines. Consequently, we cannot definitively determine whether the observed pH increases are specific to oil supplementation or represent a general effect of calorie restriction. The significant pH elevation observed in the amaranth oil group suggests a potential oil-specific effect, but the absence of control group pH data precludes definitive conclusions. Future investigations should include pH measurements across all study arms to enable comprehensive assessment of dietary intervention effects on oral physiological parameters.

Both canola and amaranth oils are high in unsaturated fatty acids, but their biochemical profiles differ [18]. The stronger effect seen with amaranth oil in this study may be due to its high squalene content and its greater antioxidant capacity. Moszak et al. demonstrated that a three-week intervention with 20 mL/day of amaranth oil led to larger improvements in fasting glucose, total cholesterol, LDL-cholesterol, and triglycerides compared to canola oil [23]. On the other hand, Kruse et al. [11] found that while four weeks of canola oil supplementation lowered fasting IL-6 and LDL-cholesterol, it was also associated with temporary post-prandial inflammation. This may explain why there were no clear differences between the two oils during our shorter intervention. Kanikowska et al. also observed different oxidative responses to the two oils, indicating that their metabolic effects may depend on the length of exposure [15]. The possible benefit of amaranth oil could be related to its higher content of squalene and polyunsaturated fatty acids, which are known to decrease NF-κB activation and cytokine production, as demonstrated by Jamka et al. [24].

Although the intervention lasted only three weeks, the observed biochemical improvements support broader evidence that even short-term dietary restriction can reduce systemic inflammation. Bianchi et al., in a meta-analysis of 76 trials, documented significant decreases in inflammatory cytokines after brief weight-loss programs. Similarly, Andriessen et al. reported improved glucose homeostasis following a three-week time-restricted feeding regimen, despite modest weight change [25,26]. Graff et al. suggested that longer interventions may lead to more pronounced adipokine modulation; however, the present findings indicate that early biochemical responses can already be detected within a relatively short intervention period [27]. The strong correspondence between salivary and systemic adipokine patterns further supports the utility of saliva as a diagnostic medium. Earlier studies have demonstrated that salivary biomarkers, including TNF-R1 and serpin A12, closely mirror systemic metabolic alterations [8,9]. Saliva offers clear advantages, non-invasive sampling, high patient acceptability, and feasibility for repeated measurements. Taken together, these findings strengthen the rationale for incorporating salivary diagnostics into obesity management and into broader oral–systemic health monitoring.

Limitations include the relatively short duration of intervention, modest sample size in subgroups, and absence of parallel plasma measurements, which limits direct comparison between salivary and systemic concentrations. Future studies should extend observation periods, incorporate blood–saliva correlations, and evaluate additional markers to strengthen diagnostic translation. Our study was not designed or adequately powered to assess sex-specific differences in treatment response. Although we have now provided sex distribution and sex-stratified baseline anthropometric data in Table 1, the small sample sizes within sex-stratified subgroups preclude reliable statistical inference regarding differential effects by sex. The unequal sex distribution across groups further complicates such analyses. Future studies should employ stratified randomization to ensure balanced sex representation and adequate statistical power to examine potential sex-specific responses to dietary interventions, as biological sex may influence adipokine metabolism, inflammatory responses, and body composition changes.

Second, anthropometric changes, while observed across all groups, were not the primary focus of our investigation and were not subjected to comprehensive statistical analysis. Our study was primarily designed to evaluate salivary biomarker responses rather than weight loss efficacy. The observed reductions in BMI and body circumferences confirm participant adherence to the calorie-restricted protocol but should be interpreted as secondary outcomes. Moreover, Important methodological limitations include the absence of concurrent serum measurements and direct adipose tissue assessment. Without paired saliva-serum samples, we cannot establish quantitative correlations or definitively confirm that salivary changes reflect systemic adipokine levels, although previous studies have demonstrated such correlations in obesity. Similarly, without imaging-based adipose tissue quantification or tissue biopsies, we cannot directly attribute salivary adipokine reductions to changes in adipose tissue mass, inflammation, or metabolic function, despite the biological plausibility of such a link given the known adipose tissue origin of these biomarkers and the observed anthropometric improvements. Future studies should incorporate simultaneous blood sampling, imaging-based adipose tissue assessment, and where feasible, adipose tissue biopsies to enable definitive mechanistic interpretation. Despite these constraints, our findings provide valuable preliminary evidence supporting the utility of salivary adipokines as non-invasive biomarkers in obesity research and suggest beneficial effects of functional oil supplementation on inflammatory markers.

These findings indicate that salivary adipokine profiling may represent a feasible and sensitive approach for monitoring metabolic inflammation and responses to dietary therapy in obesity. The reductions in salivary PAI-1, serpin A12, and TNF-R1 observed after the calorie restrictive diet reflect a favorable systemic shift toward lower inflammatory activity, with amaranth oil showing a potential additional benefit. Incorporating salivary diagnostics into clinical practice could support individualized dietary counseling, enhance adherence to lifestyle interventions, and strengthen collaboration between medical and dental professionals in managing obesity-related inflammation.

## 5. Conclusions

This three-week calorie-restricted dietary intervention, supplemented with amaranth or canola oil, demonstrated significant reductions in salivary adipocytokines (PAI-1, vaspin, and TNF-R1) in adults with obesity, suggesting favorable metabolic and anti-inflammatory effects. The observed decreases in these biomarkers indicate potential improvements in cardiovascular risk profile and systemic inflammation, highlighting saliva as a valuable, non-invasive medium for monitoring metabolic responses to dietary interventions. Additionally, the intervention improved salivary parameters, including increased flow rate and elevated pH values, which may reflect broader oral-systemic health benefits. These findings underscore the potential of functional oils rich in omega-3 and omega-6 fatty acids to modulate metabolic biomarkers and support the utility of salivary adipocytokines as accessible indicators of therapeutic efficacy in obesity management.

## Figures and Tables

**Table 1:** Baseline characteristics of study participants stratified by intervention group (mean ± SD). Subgroup A = canola oil supplementation (n = 21); Subgroup B = amaranth oil supplementation (n = 23); Control group = calorie-restricted diet without oil supplementation (n = 71). Overall comparative analysis was performed using the Kruskal–Wallis test. Sex-specific measurements are provided for descriptive purposes without statistical testing BMI, body mass index. * *p* < 0.05.

Parameter	Subgroup A (n = 21)	Subgroup B (n = 23)	Control (n = 71)	*p*-Value
Sex				
Female	10 (47.6%)	15 (65.2%)	42 (59.2%)	-
Male	11 (52.4%)	8 (34.8%)	29 (40.8%)	-
Age	41.05 ± 6.47	45.77 ± 10.81	44.89 ± 13.69	0.259
BMI (kg/m^2^)	40.99 ± 5.39	41.11 ± 7.52	42.48 ± 8.77	0.867
Waist circumference (cm)				
Overall	128.50 ± 11.01	122.09 ± 14.91	127.45 ± 21.12	0.026 *
Female	126.27 ± 11.57	115.53 ± 11.77	132.80 ± 24.39	-
Male	134.60 ± 15.61	129.88 ± 12.54	138.50 ± 12.14	-
Hip circumference (cm)				
Overall	131.59 ± 10.63	130.73 ± 15.41	133.48 ± 23.33	0.067
Female	128.41 ± 16.74	127.01 ± 16.40	131.20 ± 14.55	-
Male	127.55 ± 13.06	126.75 ± 8.31	145.00 ± 12.32	-

**Table 2:** Concentration of salivary PAI-1, TNF RI, and Serpin A12 before and after caloric restriction (mean value ± SD). The comparisons were conducted using the Mann–Whitney U test. Asterisks indicate statistically significant differences (* *p* < 0.05). Matching symbols (** *** **** *****) indicate statistically significant differences between groups for the same parameter PAI-1—plasminogen activator inhibitor-1, TNF-R1—tumor necrosis factor receptor-1.

Parameter	Subgroup	Before Caloric Restriction	After Caloric Restriction	*p*-Value
PAI-1 [pg/mL]	A	150.97 ± 116.98	127.98 ± 82.11	0.5922
	B	136.77 ± 124.38	96.17 ± 80.91	0.2361
	Control	111.01 ± 94.13	88.22 ± 72.79	0.0877
TNF RI [pg/mL]	A	542.53 ± 298.28	602.46 ± 288.91	0.4852
	B	486.02 ± 319.96 *	328.41 ± 275.05 *	0.0074
	Control	347.62 ± 266.23 **	268.17 ± 203.83 **	0.0188
Serpin A12 [pg/mL]	A	471.49 ± 696.76	363.11 ± 601.04	0.236
	B	298.89 ± 294.12 ***	173.09 ± 220.52 ***	0.0037
	Control	479.76 ± 611.66 ****	276.74 ± 373.67 *****	0.0014

**Table 3:** Salivary parameters (Mean value ± SD). The comparisons were conducted using the Mann–Whitney U test. Asterisks indicate statistically significant differences (* *p* < 0.05). Salivary pH data for the control group were not systematically recorded as part of the original study design.

Parameter	Group	Before Intervention	After Intervention	*p*-Value
Salivary flow	Subgroup A	A 0.31 ± 0.10	A 0.33 ± 0.13	0.3139
	Subgroup B	B 0.33 ± 0.19 *	B 0.39 ± 0.27 *	0.0367
	Control	0.38 ± 0.14	0.38 ± 0.14	0.6831
Salivary pH	Subgroup A	A 6.92 ± 0.47	A 7.13 ± 0.43	0.0995
	Subgroup B	B 6.74 ± 0.70	B 6.99 ± 0.49	0.3133

## Data Availability

The datasets generated and/or analyzed during the current study are available from the corresponding author upon reasonable request.

## Abbreviations

The following abbreviations are used in this manuscript:

PAI-1	Plasminogen activator inhibitor-1 (PAI-1)
TNF-R1	Tumor Necrosis Factor Receptor I
ELISA	enzyme-linked immunosorbent assay
LDL-C	low-density lipoprotein cholesterol
